# Systematic review of measurement instruments for positive development in sports: main characteristics and reported validity evidence

**DOI:** 10.1186/s41155-025-00358-x

**Published:** 2025-11-06

**Authors:** Bartira Pereira Palma, Maynara Priscila Pereira da Silva, Evandro Morais Peixoto, Ana Paula de Morais e Oliveira, Larissa Rafaela Galatti

**Affiliations:** 1 Faculdade de Educação Física, Universidade Estadual de Campinas, 701 Érico Veríssimo Av, Campinas, SP Brazil; 2 Programa de Pós-Graduação Em Psicologia, Universidade São Francisco, 105 Waldemar César da Silveira St, Campinas, SP Brazil; 3 Faculdade de Ciências Médicas, Universidade Estadual de Campinas, 126 Tessália Vieira de Camargo, Campinas, SP Brazil; 4 Faculdade de Ciências Aplicadas, Universidade Estadual de Campinas, Limeira, SP Brazil

**Keywords:** Sports psychology, Assessment, Psychometrics, Positive psychology

## Abstract

**Background:**

Positive development in sports (PDS) is a theoretical framework emphasizing human potential development in sports participation. Despite theoretical advancements, operationalizing PDS remains challenging, given the scarcity of instruments that translate theoretical models into practical applications in sports.

**Objective:**

This systematic review aimed to identify measurement instruments for assessing positive development in sports, their theoretical foundations, and validity evidence supporting their use.

**Methods:**

This study follows COSMIN guidelines and includes a comprehensive search across MEDLINE, PubMed PMC, PsycINFO, AgeLine, SPORTDiscus, CINAHL, Web of Science, and Scopus databases. The search strategy refined with expert input yielded 702 records, with 41 meeting inclusion criteria (i.e., peer-reviewed original studies focused on the development, adaptation, or validation of measurement instruments assessing positive development in sports or related constructs). Screening was performed by two researchers in a double-blind process, with conflicts resolved by a third researcher. Data extracted included sample characteristics, theoretical underpinnings, and psychometric properties.

**Results:**

Most instruments were grounded in Positive Youth Development theories and Basic Psychological Needs. Internal structure validity and internal consistency were the primary types of evidence reported, with Cronbach’s alpha widely used. Despite recognition that human potential can be developed across the lifespan, instruments primarily targeted youth in sports contexts, with limited tools for older cohorts, revealing a significant gap. Most instruments originated in high-income countries, such as those in North America and Europe, underscoring the need for adaptations of theories and tools for low- and middle-income regions.

**Conclusions:**

Underrepresentation of diverse populations with regards to race, ethnicity, and gender, absence of interpretative norms, and limited focus on older cohorts were critical limitations. Addressing these gaps can enhance PDS instruments’ inclusivity and applicability, ultimately fostering more inclusive and impactful sports practices. Furthermore, the results indicate the need to develop instruments rooted in robust PDS theoretical models alongside theoretical revisions to better represent diverse populations and people from middle- and low-income countries, in addition to the adequate adaptation of instruments.

## Introduction

Positive Development in Sport (PDS) is a theoretical approach that foresees the structuring of sport experiences to foster the development of human potential in different age groups (Dionigi et al., 2017; Rathwell & Young, 2019). PDS is derived from Positive Youth Development (PYD), which targets only adolescent and young populations, and was designed to counter programs that addressed problems commonly associated with youth, such as drug abuse and juvenile crime. These programs, which aimed to prevent negative behaviors individually, were deemed ineffective as they overlooked the co-occurrence of problem behaviors and environmental factors. Experts emphasized that merely avoiding negative behaviors was insufficient to provide youth with opportunities to fully develop their potential and enhance their chances of achieving success in life (Catalano et al., 2004). Given that sports represent a motivating activity for young people characterized by voluntary engagement, rich social interaction and opportunities to learn and practice important socio-emotional skills, it was considered an appropriate context for PYD (Larson, 2000).

Over the past two decades, PDS has become a highly discussed topic in Developmental and Sports Psychology (Dionigi et al., 2017; Fraser-Thomas et al., 2005; Gould & Carson, 2008). Bruner et al. (2022) analyzed PYD literature and categorized it into three distinct interrelated approaches. The first approach, grounded in developmental psychology, includes the Developmental Assets Framework (Benson, 1997), which identifies 40 internal and external assets fostered in sports; and the 5 Cs of PYD (Lerner et al., 2005), adapted to 4 Cs of PYD in (Côté et al., 2010), pointing to four competencies that can be developed when participants interact in sports settings. The second approach focuses on life skills development in sports, highlighting transferable skills applicable to other life domains (Weiss et al., 2016). The third, an integrative perspective, encompasses the first and second approaches, incorporating the Personal Assets Framework, emphasizing three elements—personal engagement in activities, quality relationships, and appropriate settings, that foster the 4 Cs development and leading to participation, performance, and personal development. It also includes the model proposed by Holt et al., (2017), which integrates personal and organizational factors, recognizing the influence of the relationship with different social agents in the participants’ development and the Dorsch et al., (2022) model, which considers the mutual influences between sport participants and the youth sports system.

These theoretical frameworks have not only guided conceptual understandings of positive development in sport but have also influenced the design of more robust measurement instruments. For instance, the Developmental Assets Framework inspired instruments that assess both internal characteristics (e.g., commitment to learning, positive identity) and external support (e.g., family support, safe environments), reflecting the multidimensional nature of developmental contexts in sport. The 5Cs/4Cs models have directly shaped scales that aim to capture psychological and social competencies fostered in sport participation (Vierimaa e t al., 2012; Silva et al., 2024;). The life skills approach has led to the creation of tools that measure perceived acquisition and transferability of skills such as teamwork, emotional regulation, and goal setting, emphasizing sport’s role in broader life domains (Camiré et al., 2021; Cronin & Allen, 2017; Weiss et al., 2016). Together, these frameworks have shaped a diverse and theoretically grounded set of tools for measuring positive development in sport.

Although PYD theory is well established, with several models available, recent literature shows that the field would benefit from studies designs that can demonstrate possible outcomes for young people related to engagement in sport-based PYD interventions. This includes more consistent operationalization of the frameworks and greater availability of measurement instruments (Bruner et al., 2023). In addition, PDS is now being explored across various age groups (Dionigi et al., 2017; Hawkins et al., 2009; Lerner, 2017), as the prevailing understanding is that human potential can be cultivated at any age (Bruner et al., 2022). However, the combination of stereotypes suggesting that development occurs predominantly in childhood and adolescence and the notion that older adults should only engage in light physical activities has resulted in greater scientific investment in PYD for youth. Thus, despite the current expansion in the understanding of this framework that encompasses more age ranges, there’s a need for further theory development for older cohorts, which also includes improvements in assessment strategies.

PDS is composed of latent constructs, which are unobservable variables inferred from participants’ behaviors. Thus, the only way to understand if PDS works and how is to operationalize it, that means, to derive constructs from theory that can be observed in the target population’s behavior (Razon & Tenenbaum, 2014). This enables the development of assessment tools to measure the framework so it can be studied by the academic community, providing new validity evidence and expanding the framework. Such tools also support practitioners, such as coaches and sports managers, in designing and evaluating effective interventions, thereby improving the quality of their work.

Measurement instruments are context specific (Pasquali, 2010). Professionals working in diverse sporting contexts and communities need assessment tools with validity evidence accumulated to their specific realities. PDS in general, and PYD specifically have been applied across various cultures (Qi et al., 2022). However, since PYD originated in North America, ensuring proper adaptation and validation for different contexts is critical. Additionally, as PDS is applied across diverse age ranges and settings, it is essential to determine the availability of appropriate instruments, the evidence supporting their use, and the constructs they assess. Mapping existing measurement instruments is vital to advancing the field scientifically and assisting practitioners in selecting suitable tools. The aim of this systematic review is to identify measurement instruments of positive development in sports, the theoretical bases underlying their construction, and the validity evidence supporting their use. This review is guided by the following questions: what are the available measurement instruments to assess constructs related to positive development in sports, and for what demographics they are suitable? What are the theoretical frameworks that underpin these instruments? Do these instruments accumulate the minimum validity evidence supporting their use? What are the instruments’ main psychometric analyses and properties reported?

## Methods

### Procedures

We conducted a search on the Prospero website (https://www.crd.york.ac.uk/prospero/) to identify registered systematic review protocols similar to ours, and found review focusing on instruments that measure life skills in sports. After discovering this study was unpublished, we reviewed the doctoral dissertation from which it was derived and confirmed its focus differed from ours, as it emphasized identifying studies on life skills assessment strategies, age groups, and psychological variables rather than the instruments themselves. Consequently, we registered our protocol on Prospero’s database. Following the COnsensus-based Standards for the selection of health Measurement Instruments (COSMIN-https://www.cosmin.nl) guidelines (Munn et al., 2018) we adapted the criteria to better suit our research aims. For example, we included instruments even if they had only the content validity since our objective was to gather existing instruments and available evidence of validity to contribute to the process of instrument selection by professionals in the field and to highlight areas lacking future studies. It was not our objective to identify the most appropriate instrument, as the appropriateness of an instrument is inherently context-dependent. Thus, we did not employ the COSMIN risk of bias tool, although we reported the available validity evidence of the instruments included in the review and assessed the quality of the studies based on specialized literature (American Educational Research Association et al., 2014; Terwee et al., 2018). The Ethics Committee of the University of xxx approved this study (number: 50513321.4.0000.5404).

### Search strategy

The librarian of the Faculty of Medical Sciences of the University of Campinas guided the development, testing, and refinement of the search strategy over six months. Meetings were held weekly during the first month, bi-weekly in the second, and monthly thereafter, with additional discussions as needed. Searches were conducted across eight databases: MEDLINE (via PubMed), PubMed PMC, PsycINFO, AgeLine, SPORTDiscus, CINAHL (via EBSCOhost), Web of Science, and Scopus. Additional articles were identified through back-referencing.

A preliminary search identified key terms frequently used in studies on this topic. These terms were expanded using controlled vocabulary in each database (e.g. Thesaurus, Medical Subject Headings), meaning that search strategies were tailored to each database, because different terms with similar meanings are used in different controlled vocabularies. Keywords were organized into four thematic groups:

1. Positive development, competence, life skills, social skills.
2. Measurement instruments, tests, scales.
3. Psychometrics, validity, instrument development, instrument construction.
4. Sports, athletes, sport, and exercise measure.

Terms within each group were combined using the Boolean operator OR. Searches were conducted in each data base by inputting Group 1 AND Group 2 AND Group 3 AND Group 4. The strategy was refined through multiple iterations, using a selected article as a benchmark to ensure accuracy. Reference lists of included articles were manually reviewed, yielding four additional articles. The final search strategy for PsycINFO is provided in Table 1..

**Table 1:** Search strategy constructed for the PsycINFO database

((IndexTermsFilt: (“Sport and Exercise Measures”) OR IndexTermsFilt: (“Sports”) OR IndexTermsFilt: (“Sport Psychology”) OR IndexTermsFilt: (“Physical Activity”) OR IndexTermsFilt: (“Exercise”) OR IndexTermsFilt: (“Athletes”) OR IndexTermsFilt: (“Adaptive Sports”)) OR (abstract: (“Sport and Exercise Measures”) OR abstract: (Sports) OR abstract: (“Sport Psychology”) OR abstract: (“Physical Activity”) OR abstract: (Exercise) OR abstract: (Athletes) OR abstract: (“Adaptive Sports”)) OR (title: (“Sport and Exercise Measures”) OR title: (Sports) OR title: (“Sport Psychology”) OR title: (“Physical Activity”) OR title: (Exercise) OR title: (Athletes) OR title: (“Adaptive Sports”))) AND ((IndexTermsFilt: (“Competence”) OR IndexTermsFilt: (“Social Skills”) OR IndexTermsFilt: (“Life Skills”) OR IndexTermsFilt: (“Psychosocial Assessment”) OR IndexTermsFilt: (“Psychosocial Development”) OR IndexTermsFilt: (“Psychosocial Factors”)) OR (abstract: (Competence) OR abstract: (“Social Skills”) OR abstract: (“Life Skills”) OR abstract: (“Psychosocial Assessment”) OR abstract: (“Psychosocial Development”) OR abstract: (“Psychosocial Factors”) OR abstract: (“positive development”) OR abstract: (“positive youth development”) OR abstract: (“sport values”)) OR (title: (Competence) OR title: (“Social Skills”) OR title: (“Life Skills”) OR title:
(“Psychosocial Assessment”) OR title: (“Psychosocial Development”) OR title: (“Psychosocial Factors”) OR title: (“positive development”) OR title: (“positive youth development”) OR title: (“sport values”))) AND ((IndexTermsFilt: (“Surveys”) OR IndexTermsFilt: (“Questionnaires”) OR IndexTermsFilt: (“Likert Scales”) OR IndexTermsFilt: (“Instrumentality”) OR IndexTermsFilt: (“Measurement”) OR IndexTermsFilt: (“Test Battery”)) OR (title: (Surveys) OR title: (Questionnaires) OR title: (“Likert Scales”) OR title: (Instrumentality) OR title: (Measurement) OR title: (“Test Battery”)) OR (abstract: (Surveys) OR abstract: (Questionnaires) OR abstract: (“Likert Scales”) OR abstract: (Instrumentality) OR abstract: (Measurement) OR abstract: (“Test Battery”))) AND ((IndexTermsFilt:(“Psychometrics”) OR IndexTermsFilt:(“Statistical Validity”) OR IndexTermsFilt:(“Test Validity”) OR IndexTermsFilt:(“Test Reliability”) OR IndexTermsFilt:(“Test-Retest Reliability”)) OR (Title:(“Psychometrics”) OR Title:(“Statistical Validity”) OR Title:(“Test Validity”) OR Title:(“Test Reliability”) OR Title:(“Test-Retest Reliability”) OR Title:(“validity evidence”) OR Title:(“instrument development”) OR Title:(“instrument construction”)) OR Abstract:(Psychometrics” OR “Statistical Validity” OR “Test Validity” OR “Test Reliability” OR “Test-Retest Reliability” OR “validity evidence” OR “instrument development” OR “instrument construction”))

### Inclusion and exclusion criteria

The present review inclusion criteria were (1) original research articles published in peer-reviewed journals; (2) studies on the development, adaptation, or accumulation of validity evidence of measurement instruments assessing positive development in sports or related constructs in any age range; (3) studies in which the measurement instrument is the object of the study and not used to assess positive development as a dependent variable; (4) studies that assessed at least one validity evidence of the instrument that is object of the study; (5) articles published from 1989 onwards were included, as this period marks the onset of broader discussions and emerging approaches to positive development in the literature (Catalano et al., 2004).

The exclusion criteria were: (1) studies published in abstract format, book chapters, thesis, or dissertations; (2) studies in languages other than Portuguese, English, Spanish, and French were reported but excluded because these four are the languages the researchers are fluent in reading; (3) studies on other topics.

### Screening

The final search yielded 707 records. The records were uploaded onto an online reference management platform (Ouzzani et al., 2016). Duplicates were identified by the platform and manually deleted. Three researchers, experts in positive development in sports, participated in the screening phase. The screening was performed by two Ph.D. candidates and a professor holding a Ph.D. degree. In the first stage, titles, keywords, and abstracts of the remaining records after the removal of duplicates were then screened by two independent researchers who were blind to each other. The articles that met the criteria were included in a second screening stage in which they were read in their entirety by the two Ph.D. candidates, also conducted in a double-blind approach to assess the eligibility criteria. In both stages conflicts were solved by the professor, who was blind to the previous researchers’ decisions. The stages of the screening phases were conducted on the online platform, which has a tool to inform the exclusion reasons.

### Data analysis and quality assessment

Data were analyzed descriptively. The quality of each instrument included in the review was assessed by analyzing the procedures and results of the validity evidence estimated. Good quality content validity was considered present when the researchers consulted experts and the target population when constructing or adapting the items (Terwee et al., 2018). The quality of the results of the studies estimating validity evidence based on internal structure and reliability was assessed considering internationally employed standards (American Educational Research Association et al., 2014).

### Data extraction process

A data extraction spreadsheet was constructed by the researchers. We collected the following information from the included studies: authors and year of publication, name of the instrument object of the study, method (development, transcultural adaptation, or accumulation of validity evidence), country of origin, theoretical framework of the instrument, sample characteristics, type of validity evidence tested, and estimated indexes, factors description, quantity of items. Two reviewers, blind to each other, extracted the data independently. Discrepancies were resolved through discussion, and consensus was reached among the reviewers. Reviewers were not blind to the authors, institutions, or journal titles in this step of the review.

## Results

A total of 41 studies met the inclusion criteria and were analyzed. Five of these were identified through a manual search (i.e., suggested by publisher websites during full-text downloads or found in the reference lists of previously included articles). Figure 1 presents the study selection flowchart.

Extracted data are presented in Table 2, which details study nationality, study type, theoretical background of the instruments, and content validity quality. Table 3 summarizes other psychometric properties.

**Table 2:** Main characteristics and content validity sample of the measurement instruments included in the systematic review

Reference	Name of the instrument	Constructs measured	Content validity sample characteristics	Design	Country
Aguilar and Petrakis (1989)	Perceived competence and satisfaction in racquet sports	Perceived competence and satisfaction for racquet sports	62 athletes (target population)/5 experts	CO	USA
Albouza et al. (2021)	Youth Sport Values Questionnaire-2	Values in sports	20 athletes (10 W, 14.31 ± 1.70 YO–target population), 2 translators	TA	France
Alexe et al. (2022)	Need Satisfaction and Frustration Scale	Need satisfaction and frustration	4 translators, 2 experts, 11 athletes (target population)	AD	Romania
Aouani et al. (2019)	Profile of Emotional Competence	Emotional competence	Experts, 20–30 subjects (target population), 2 translators	TA	Tunisia
Bean et al. (2018)	Program Quality Assessment in Youth Sport	Sport program quality	32 experts, 34 coaches (target population)	CO	Canada
Bean et al. (2020)	Learning Climate Questionnaire	Learning climate	N/A	AD	Canada
Bhavsar et al. (2020)	Psychological Need States in Sport-Scale	Satisfaction, frustration, and unfulfillment of psychological needs	1 researcher, 2 senior researchers, and research team	CO	Australia
Camiré et al. (2021)	Coaching life skills in sport questionnaire	The extent to which coaches teach life skills through sports	6 professors (experts), 26 high school coaches (target population)	CO	Canada
Costa et al. (2017)	Basic Psychological Needs in Exercise Scale in Physical Education	Basic psychological needs	3 professors (experts), 3 translators	AD	Brazil
Cronin and Allen (2017)	Life Skills Scale for Sport	Life skills	39 researchers (experts), Flesch-Kincaid readability assessment	CO	United Kingdom
Emm-Collison et al. (2016)	Adolescent Psychological Need Support in Exercise Questionnaire	Psychological need support in exercise	7 academics (experts)	CO	United Kingdom
Francisco et al. (2018)	Basic Needs Satisfaction in Sport Scale—Spanish version	Basic needs satisfaction in sports	2 translators, 5 sport psychologists (experts)	TA	Spain
Gonçalves et al., (2017)	Youth Sports Values Questionnaire	Sport values	10 youth (11 YO), 5 adults sport practitioners, 5 highly educated adults (target population), 3 researchers, and 1 translator (translators)	TA	Brazil
González-Cutre et al. (2015)	Escala de Satisfacción de las Necesidades Psicológicas Básicas en General	Satisfaction of basic psychological needs	Group of experts in motivation (experts), small group of adults (target population), translators	AD	Spain
Gunnell et al. (2012)	Psychological Need Satisfaction in Exercise Scale	Psychological need satisfaction in exercise	N/A	AD	Canada
Gutiérrez Marín et al. (2017)	Cuestionario de Conducta Apropiada en la Educación Física y el Deporte	Condutas apropriadas na educação física e no esporte	4 researchers (experts)	CO	Spain
Laffrey and Asawachaisuwikrom (2001)	The exercise self-efficacy questionnaire	Self-efficacy in exercise	7 nurses working with the target population (experts)	CO	USA (Mexican women)
Lee et al. (2008)	Youth Sport Values Questionnaire	Values in sports	Not reported	AD	United Kingdom
Liu and Chung, (2014)	Psychological Needs Satisfaction Scale in Physical Education	Psychological need satisfaction in physical education	3 academics and 7 physical education teachers (experts), 26 students (target population)	CO	China
MacDonald et al. (2012)	Youth Experience Survey for Sport	Positive and negative experiences in sports	5 youth sport researchers (experts)	AD	United Kingdom/Canada
Manzo et al. (2001)	Carolina Sport Confidence Inventory	Sport confidence	5 psychologists and sport psychologists (experts)	CO	USA
Mossman et al. (2021)	Life Skills Scale for Sport–Transfer Scale	Life skills	10 researchers–5 M and 5 W (experts), 72 sports participants (21.10 ± 3.18 YO–target population)	CO	United Kingdom
Myers et al. (2006)	Coaching Competency Scale	Coaching competence	9 coaches (experts)	CO	USA
Nascimento-Junior et al. (2020)	Portuguese Version of the Life Skills Scale for Sport	Life skills	4 translators, 5 academics (experts), 25 sport participants (13–18 YO, target population)	TA	Brazil
Ng et al. (2011)	Basic Needs Satisfaction in Sport Scale	Basic needs satisfaction in sports	10 researchers (experts), 4 translators	CO	China/New Zealand
Ogawa and Neves (2020)	Sport Character Scale–Brazilian version	Character in sports	8 athletes (target population), 6 experts, 4 translators	TA	Brazil
Rathwell and Young (2016)	University Sport Experience Survey	Positive development in sport	6 researchers (experts), 14 university athletes (target population)	AD	Canada
Rathwell et al. (2021)	University Sport Experience Scale	Positive development in sport	Translation (4 translators and 3 experts). Content validity: 20 university athletes (between 18 and 25 YO, 12 men) and 3 experts	TA	Brazil
Sabourin et al. (2020)	Short Form Youth Experiences Survey/Sport and Life Skills Scale for Sport	Life skills/positive and negative youth sport experiences	4 translators, 7 researchers (experts), 137 student athletes (target population)	TA	Canada
Saunders et al. (1997)	The Social Influences scale, The Self-Efficacy scale, The Beliefs scale	Psychosocial influences on children's physical activity	Fifth-grade students (target population)	AD	USA
Stuntz and Spearance (2010)	Cross-domain relationship measure	Cross-domain relationships	5 current and former athletes, coaches, and researchers (experts)	CO	USA
Sturm et al. (2020)	Needs Satisfaction in Physical Education Scale (German version)	Basic psychological needs in physical education	Translators	AD	Germany
Vlachopoulos and Michailidou (2006)	The Basic Psychological Needs in Exercise Scale	Basic psychological needs in exercise	3 experts, 20 exercise participants (target population)	CO	Greece
Vlachopoulos (2007)	Basic Psychological Needs in Exercise Scale in Community Exercise Programs	Basic psychological needs in exercise	N/A	VA	Greece
Vlachopoulos (2008)	Basic Psychological Needs in Exercise Scale in Community Exercise Programs	Basic psychological needs in exercise	N/A	AD	Greece
Vlachopoulos et al. (2013)	Basic Psychological Needs in Exercise Scale	Basic psychological needs in exercise	Translators	CI	Greece/Portugal/Spain/Turkey
Wang et al. (2017)	Physical activity self-efficacy scale	Physical activity self-efficacy	N/A	AD	China
Watanabe et al. (2017)	Physical Activity Self-Regulation scale– Japanese version	Physical activity self-regulation	3 translators, 5 experts	TA	Japan
Weiss and Smith (1999)	Sport Friendship Quality Scale	Quality of youth sport friendships	Children and adolescents (target population), 7 experts	CO	USA
Weiss et al. (2014)	Life Skills Transfer Survey	Life skills transfer	11 experts, 31 adolescents, boys and girls (target population)	CO	USA
Wilson et al. (2006)	The Psychological Need Satisfaction in Exercise Scale	Psychological need satisfaction in exercise	40 experts	CO	Canada

*YO* years old, *W* women, *M* men, *N/A* not applicable, *CO* construction, *TA* transcultural adaptation, *AD* adaptation, *CI* cross-cultural invariance, *VA* validity evidence accumulation

**Table 3:** Psychometric properties of the instruments included in the systematic review

Reference	Instrument	Sample	Psychometric properties
Aguilar and Petrakis (1989)	Perceived Competence and Satisfaction in Racquet Sports	Internal structure: first run-163 students (gender not reported). Second run–208 (127 men, 78 women, 18–74 YO)	Internal structure: PCA-Two-factor solution for the satisfaction measure (sport enjoyment and leisure satisfaction); one-factor solution for the competence measure Reliability: Cronbach’s α–competence scale: 0.90; satisfaction scale: 0.77
Albouza et al. (2021)	Youth Sport Values Questionnaire-2 (YSVQ-2)	Internal structure and invariance: 522 athletes (mage = 16.07 SD = 2.21, 265 boys, individual sports *n* = 305, team sports *n* = 215)	Internal structure: CFA for each subscale–moral (CFI = 0.99; TLI = 0.99, RMSEA = 0.02), competence (CFI = 0.98; TLI = 0.94; RMSEA = 0.08), and status (CFI = 0.97; TLI = 0.90; RMSEA = 0.08) Invariance: MGCFA–configural and metrical invariances were demonstrated for sex. Configural, metric, and scalar invariance were demonstrated for type of sport (for individual vs team sport), and for application time (time 1 vs time 2) Reliability: Cronbach’s α–moral: 0.76; competence: 0.74; status: 0.79
Alexe et al. (2022)	Need Satisfaction and Frustration Scale	Internal structure and invariance: 642 athletes (mage = 22.81, SD = 5.78, 354 men, individual sports *n* = 305, team sports *n* = 337)	Internal structure: CFA–6 factors (CFI = 0.967; TLI 0.955; SRMR = 0.042; RMSEA = 0.042) Invariance: MGCFA–there was support for configural, metrical and scalar invariance across gender, age, and sport Reliability: Cronbach’s α–between 0.75 and 0.89. Raykov–0.76 to 0.89
Aouani et al. (2019)	Profile of Emotional Competence	Internal structure: 285 adolescents (mage = 15.2, 153 boys, 101 athletes and 184 non-athletes)	Internal structure: EFA: 2-factor solution Reliability: Cronbach’s α–For all the questionnaires: 0.859 (0.858 adjusted); interpersonal competence 0.688 (0.686 adjusted); intrapersonal competence 0.750 (0.752 adjusted) Test–retest–ICC: interpersonal competencies (range = 0.94 to 0.99) and intrapersonal competencies (range = 0.96 to 0.98). For the interpersonal domain the ICC was 0.98 (95% CI 0.9776–0.9860), for the intrapersonal domain it was 0.98 (95% CI 0.9802–50.9876), and for the general questionnaire it was 0.99 (95% CI 06.9830–0.9893)
Bhavsar et al. (2020)	Psychological Need States in Sport-Scale	Study 1–Internal structure and discriminant validity: 301 competitive athletes (mage = 20.27, SD = 7.36, 209 women) Study 2–Internal structure: 333 competitive athletes (mage = 19.99, SD = 5.43, 183 women)	Internal structure: Study 1–CFA and ESEM–12 concurrent models were tested. The results indicated 6-factor solution: ESEM-*χ*^2^ (99) = 171.110, *p* < 0.001, CFI = 0.97, TLI = 0.94, SRMR = 0.02, RMSEA = 0.05 (90%, CI = 0.04, 0.06) Study 2–ESEM–*χ*^2^ (247) = 438.72, *p* < 0.001, CFI = 0.97, TLI = 0.95, SRMR = 0.02, RMSEA = 0.05 (90%, CI = 0.04, 0.06) Convergent/discriminant validity: SEM–autonomy support predicted autonomy satisfaction (*β* = 0.74), competence satisfaction (*β* = 0.25), and relatedness satisfaction (*β* = 0.18); competence support predicted competence satisfaction (*β* = 0.33); relatedness support predicted autonomy satisfaction (*β* = 0.19), competence satisfaction (*β* = 0.32), and relatedness satisfaction (*β* = 0.64). Autonomy satisfaction predicted dedication (*β* = 0.42), and positive affect (*β* = 0.012); competence satisfaction predicted dedication (*β* = 0.33) and positive affect (*β* = 0.43); relatedness satisfaction predicted positive affect (*β* = 0.30). Autonomy thwarting predicted autonomy frustration (*β* = 0.76) and competence frustration (*β* = 0.03); competence thwarting predicted competence frustration (*β* = 0.79) and relatedness frustration (*β* = 0.49); relatedness thwarting predicted autonomy frustration (*β* = 0.25), relatedness frustration (*β* = 0.45), and competence frustration (*β* = 0.13). Autonomy frustration predicted exhaustion (*β* = 0.26) and negative affect (*β* = 0.24); competence frustration predicted exhaustion (*β* = 0.47) and negative affect (*β* = 0.42); relatedness frustration predicted negative affect (*β* = 0.24). All *p* < 0.05 Internal consistency: Raykov’s rho–between 0.52 and 0.78
Bean et al. (2018)	Program Quality Assessment in Youth Sport (PQAYS)	Convergent validity: 22 young athletes (mage = 13.66, SD = 2.91, 161 boys)	Reliability: Cronbach’s α–In 8 of the 10 subscales *α* > 0.70; in 2 subscales (physical safety and opportunity to belong) *α* < 0.70; for the whole instrument *α* = 0.84 Inter-rater reliability: for the total measure (*κ* = 75; [*p* <.0005], 95% confidence interval [0.74, 0.76]) and for each subscale (κrange = 0.6–0.88)
Bean et al. (2020)	Learning Climate Questionnaire	Study 1: 445 youth athletes (mage = 14.25, SD = 2.42, 209 boys, 1 gender fluid Study 2: 253 youth athletes (mage = 13.70 SD = 2.36, 124 boys)	Internal structure: CFA–the 15-item version showed more adequate indexes than the 24-item version (CFI = 0.96, TLI = 0.95, RMSEA = 0.05, 90% CI [0.04, 0.06], SRMR = 0.04, AICc = 17,430.48) Reliability: Cronbach’s α–ranged from 0.82 to 0.90 for all subscales Invariance: MGCFA–there was support for configural, metrical and scalar invariance across sex
Camiré et al. (2021)	Coaching life skills in sport questionnaire	Internal structure: exploratory sample (*n* = 623 coaches, Mage = 40.4 SD = 12.0, 391 men). Confirmatory sample (*n* = 817, Mage = 43.9, SD = 11.7, 525 men)	Internal structure: ESEM–5-factor solution, 38 items with 2 problematic items (CFI = 0.948, TLI = 0.931, SRMR = 0.026, RMSEA = 0.043 (90% CI [0.039, 0.046]) and *χ*^2^/df = 2.150) Confirmatory ESEM–5 factors and 36 items (CFI = 0.932, TLI = 0.908, SRMR = 0.026, RMSEA = 0.048 (90% CI [0.045, 0.052]) and *χ*^2^/df = 2.919) Invariance: MGESEM–there was support for configural, metrical and scalar invariance across gender, years of coaching experience, and coaching education Reliability: Cronbach’s α-All *α* > 0.70 (*r* = 0.89–0.93)
Costa et al (2017)	Questionário de Necessidades Psicológicas no Exercício (BPNES)	Internal structure: 403 students (mage = 14.30, SD = 1.53, 204 boys)	Internal structure: CFA–3-factor solution (*x*^2^/df = 2.103; CFI = 0.962; GFI = 0.959; TLI = 0.951; RMSEA = 0.052; [RMSEA ≤ 0.05] = 0.370) Reliability: Cronbach’s α–General scale: *α* = 0.83. Factors-autonomy: 0.66, competence: 0.80, relationship: 0.80. Composite reliability: autonomy–0.66, competence–0.8, relationship: 0.81 Convergent validity: AVE–autonomy: 0.33; competence: 0.52; relationship: 0.51
Cronin and Allen (2017)	Life Skills Scale for Sport	Internal structure: exploratory sample (338 youth sports participants, Mage = 14.71, SD = 2.42, 189 boys); confirmatory sample (223 youth sports participants, Mage = 15.01, SD = 2.81, 131 boys); test–retest sample: (37 youth sports participants, Mage = 18.96, SD = 1.25, 24 boys)	Internal structure: EFA–4-factor structure for the teamwork subscale with eigenvalues > 1.0, but the PA suggested the retention of 2 factors. To the other life skills, even though some eigenvalues suggested additional factors, the scree plots and parallel analysis suggested retaining one factor CFA-general 8-factor model (CFI = 0.91; TLI = 0.90; RMSEA = 0.05). The ESEM indexes were more adequate (CFI = 0.95; TLI = 0.93; RMSEA = 0.04) Reliability: Cronbach’s α-*α* > 0.70 for all subscales Test–retest: ICC–teamwork: 0.93, goal setting: 0.93, time management: 0.92, emotional skills: 0.87, communication: 0.89, social skills: 0.86, leadership: 0.93, problem solving and decision making: 0.82
Emm-Collison et al. (2016)	Adolescent Psychological Need Support in Exercise Questionnaire	Internal structure: 443 adolescents (mage = 13.74, SD = 0.76, 211 boys)	Internal structure: CFA–3-factor solution (autonomy, competence, and relatedness) applied to three social agents–family (CFI = 0.99; TLI = 0.99; WRMR = 0.62; RMSEA = 0.08); friends (CFI = 0.99; TLI = 0.99; WRMR = 0,75; RMSEA = 0.09); physical education teacher (CFI = 0.99; TLI = 0.98; WRMR = 0.88; RMSEA = 0.14) Reliability: Cronbach’s α–ranging from 0.77 to 0.93
Francisco et al. (2018)	Basic Needs Satisfaction in Sport Scale	Internal structure: 441 team sports athletes (Mage = 17.46, SD = 3.59, 53.5% women)	Internal structure: CFA–5-factor solution [χ 2 (499.68)/df (160) = 3.13; RMSEA = 0.06 (IC 90%; 0.06–0.07); NNFI = 0.96; CFI = 0.97] Invariance: MGCFA–there was support for configural, metrical and scalar invariance across sex and age Reliability: Cronbach’s α–competence: 0.88; autonomy-choice: 0.95; autonomy IPLOC: 0.92; autonomy-volition: 0.82; relatedness: 0.95
Gonçalves et al. (2017)	Youth Sport Values Questionnaire-2 (ysvq-2)	Internal structure: PCA (18 youth athletes (Mage = 16.54, SD = 3.53, 114 girls); CFA (200 youth athletes, Mage = 24.14, SD = 8.89, 50 women)	Internal structure: EFA–3-factor solution CFA-3-factors solution [*χ*^2^(148) = 108.43, *p* < 0.001, *χ*^2^/gl = 2.26, GFI = 0.92, CFI = 0.90, RMSEA = 0.08 (CI 90% = 0.06–0.10)] Reliability: Cronbach’s α–EFA sample (moral: 0.70; status: 0.63; competence: 0.57); CFA sample (moral: 0.75; status: 0.73; competence: 0.67)
González-Cutre et al. (2015)	Escala de Satisfacción de las Necesidades Psicológicas Básicas en General	Internal structure and invariance: 399 (Mage: 31.30, SD = 11.31, 202 men)	Internal structure: CFA–3-factor solution (*χ*^2^ (96, *N* = 399) = 181.96, *p* < 0.001; *χ*^2^/df = 1.89; CFI = 0.92; IFI = 0.92; RMSEA = 0.047 (90% CI = 0,037–0.058); SRMR = 0.048) Invariance: MGCFA–for sex and age–there was support for configural, metrical, and scalar invariance Reliability: Cronbach’s α–autonomy: 0.81; competence: 0.72; relationship: 0.82
Gunnel et al. (2012)	Psychological Need Satisfaction in Exercise Scale	Internal structure and invariance: university students/exercisers (30 men, Mage = 32.03, SD = 1.322; 253 women, Mage = 26.13, SD = 8.56); general population (67 men, Mage = 40.27, SD = 21.01, 146 women, Mage = 34.07, SD = 18.34); people with osteoporosis (28 men, Mage = 62.89, SD = 14.68; 192 women, Mage = 67.92, SD = 11.11)	Internal structure: CFA–3-factor solution–Sample that answered the original version (S–B*χ*^2^ = 270.72, *p* < 0.001; CFI = 0.99; RMSEA = 0.06, CI 90% (IC) = 0.05–0.07); sample that answered the modified version (S– B*χ*^2^ = 343.58, *p* < 0.001; CFI = 0.98; RMSEA = 0.08, 90% CI = 0.08–0.10); sample with osteoporosis (S–B*χ*^2^ = 262.48 *p* < 0.001; CFI = 0.99; RMSEA = 0.07, CI 90% = 0.06–0.08) Invariance: MGCFA–configural and metric invariance was supported for three different samples (physical exercise participants, general population, people with osteoporosis) Reliability: Composite reliability and average variance extracted–ρc ≥ 0.94 e ρv ≥ 0.72 for sample 1, ρc ≥ 0.95 and ρv ≥ 0.77 for sample 2, and ρc ≥ 0.95 e ρv ≥ 0.75 for sample 3
Gutiérrez Marín et al. (2017)	Cuestionario de Conducta Apropiada en la Educación Física y el Deporte	Internal structure: 352 students in the 6th year of elementary school (179 girls)	Internal structure: EFA–10-factor solution Reliability: Cronbach’s α–appropriate skills to lose: 0.19; appropriate skills to win: 0.43; appropriate skills during the game: 0.54; fair play skills: 0.56; social skills: 0.56
Laffrey and Asawachaisuwikrom (2001)	The exercise self-efficacy questionnaire	Internal structure: 77 women (from 60 to 87 YO)	Internal structure: EFA–1-factor solution
Lee et al. (2008)	Youth Sport Values Questionnaire	Internal structure: Study 1–491 school or club competitors (258 males) (Mage = 13.42, SD = 1.03); Study 2–892 sports practitioners (503 men) (Mage = 13.89, SD = 1.05)	Internal structure: CFA–3-factor solution (CFI = 0.95; NNFI = 0.94; RMSEA = 0.05; SRMR = 0.05) Invariance: MGCFA–there was support for configural, metrical, and scalar invariance across sex Reliability: Cronbach’s α–moral values: 0.70; competence values: 0.74; status: 0.82
Liu and Chung (2014)	Psychological Needs Satisfaction Scale in Physical Education	Internal structure: 646 students (Mage = 13.67, SD = 1.06, 348 boys) Psychometric properties: 563 junior secondary school students (Mage = 13.63, SD = 1.02, 264 men)	Internal structure: EFA–3-factor solution CFA–3-factor solution [*χ*^2^ (32) = 95.88, *p* < 0.001, CFI = 0.977, SRMR = 0.03, RMSEA = 0.06 (90% CI: 0.05–0.07)] Reliability: Cronbach’s α–ranged from 0.82 to 0.84 Test–retest: ICC–autonomy satisfaction (M1 = 4.63; SD1 = 0.97; M2 = 4.50; SD2 = 1.08), *r* = 0.83 (CI 95%: 0.73–0.88); competence satisfaction (M1 = 4.41; SD1 = 1.17; M2 = 4.36; SD2 = 1.34), *r* = 0.85 (CI 95%: 0.77–0.90); e relatedness satisfaction (M1 = 5.17; SD1 = 1.25; M2 = 4.94; SD2 = 1.23), *r* = 0.80 (95% CI: 0.68–0.87)
MacDonald et al. (2012)	Youth Experience Survey	Internal structure: 637 athletes (Mage = 15.00, SD = 1.5, 333 boys)	Internal structure: CFA–11 concurrent models were tested, which presented inadequate fit indices or in disagreement with the theoretical framework EFA–5-factor solution Reliability: Cronbach’s α–between 0.82 and 0.92
Manzo et al. (2001)	Carolina Sport Confidence Inventory (CSCI)	First internal structure and psychometric properties: 293 university students (from 18 to 43 YO, 124 men, 5 unknown). Further validity evidence: 123 undergraduate varsity athletes (46 men, from 18 to 23 YO)	Internal structure: EFA–2-factor solution CFA–2-factors solution (significant chi-square values (*X*^2^, 78, *N* = 123) = 558.54, *p* < 0.05 ESEM-Bentler-Bonnett NNFI: 0.83, CFI: 0.86 Convergent validity: 13-item CSCI with TSCI (*r* = 0.66, *p* = 0.01, *r*^2^ = 0.43), 13-item CSCI with self-confidence subcomponent of CSAI-2 (*r* = 0.57, *p* = 0.01, *r*^2^ = 0.32), 13-item CSCI with PPSS (*r* = 0.46, *p* = 0.001, *r*^2^ = 0.21), 13-item CSCI with TRS (*r* = 0.33, *p* = 0.001, *r*^2^ = 0.11) Reliability: Cronbach’s α–General scale: 0.92; perceived competence: 0.92; dispositional optimism: 0.86 Test–retest: ICC–13-item CSCI: 0.94, competence factor: 0.94, dispositional optimism: 0.78
Mossman et al. (2021)	Life Skills Scale for Sport–Transfer Scale (LSSS-TS)	Internal structure: 321 youth sports participants (mage = 14.20, SD = 1.07, 266 boys)	Internal structure: CFA–8-factor solution (CFI = 0.94; TLI = 0.93; RMSEA = 0.05). Bifactor–CFI = (0.94; TLI = 0.93; RMSEA = 0.05) ESEM: CFI = (0.96; TLI = 0.93; RMSEA = 0.05) ESEM Bayesian: (CFI = 0.97; TLI = 0.94; RMSEA = 0.05) Reliability: Cronbach’s α–teamwork: 0.84; goal setting: 0.87; social skills: 0.87; problem solving and decision making: 0.87; emotional skills: 0.89; leadership: 0.90; time management: 0.89; interpersonal communication: 0.90; and total life skills transfer: 0.97
Myers et al. (2006)	Coaching Competency Scale (CCS)	Internal structure: 407 soccer players (165 men) and 183 ice hockey players (gender not reported) Mage = 19.53, SD = 1.34	Internal structure: CFA–4-factor solution-(*χ*^2^ (246) = 1266.42, *p* < 0.001, *χ*^2^/df = 5.15, CFI = 0.91, TLI = 0.90, SRMR = 0.05 e RMSEA = 0.09) Reliability: Cronbach’s α–motivation competence: 0.90, game strategy competence: 0.87, technique competence: 0.85, character building competence: 0.82
Nascimento-Junior et al (2020)	Portuguese Version of the Life Skills Scale for Sport	Internal structure: 413 youth athletes (Mage = 16.27, SD = 3.33, 277 boys) Convergent validity: 134 youth athletes (Mage = 13.62, SD = 1.23, 71 boys)	Internal structure: CFA–8 correlated-factor model–*χ*^2^ (832) = 1180.54, CFI = 0.94, TLI = 0.93, RMSEA = 0.03 Bifactor model–*χ*^2^ (817) = 1161.17, CFI = 0.94, TLI = 0.93, RMSEA = 0.03 Reliability: Cronbach’s α and composite reliability, respectively–teamwork (0.76, 0.77), goal setting (0.85, 0.85), social skills (0.77, 0.78), problem solving and decision making (0.79, 0.79), emotional skills (0.78, 0.78), leadership (0.87, 0.87), time management (0.81, 0.82), interpersonal communication skills (0.79, 0.79), and total life skills (0.95, 0.97) Convergent validity: Pearson’s correlation–the eight life skills and total life skills showed significant and positive correlations with intrinsic motivation, integrated regulation, and identified regulation (*r*’s between 0.14 and 0.33); exception for goal setting (no significant relationship with intrinsic motivation–*r* = 0.08). No significant relationships between the eight life skills/total life skills and introjected regulation, external regulation, and amotivation; exception for negative associations between interpersonal communication skills and external regulation (*r* = − 0.17), and social skills and external regulation (*r* = − 0.12)
Ng et al. (2011)	Basic Needs Satisfaction in Sport Scale (BNSSS)	Internal structure: Study 1–273 Chinese athletes (Mage = 20.75, SD = 1.64, 140 women) Study 2–401 New Zealand athletes (Mage = 18.97, SD = 1.95, 237 women). Test–retest reliability: 63 Chinese athletes (Mage = 21.22, SD = 1.96, 25 women)	Internal structure: China sample–CFA–3-factor solution–15 items (NNFI = 0.98; CFI = 0.98; SRMR = 0.05; RMSEA = 0.06). New Zealand sample CFA–5-factors solution–20 items (NNFI = 0.96; CFI = 0.97; SRMR = 0.07; RMSEA = 0.06) Reliability: Cronbach’s α–China sample–competence: 0.87; autonomy: 0.83; relatedness: 0.80. New Zealand sample–BNSS competence: 0.77; BNSS choice: 0.85; BNSS IPLOC: 0.76; BNSS volition: 0.61, BNSS: relatedness 0.77 Test–retest: ICC–New Zealand sample CFA–5-factors solution–Competence = 0.83, Choice = 0.78, IPLOC = 0.87, Volition = 0.83, Relatedness = 0.74
Ogawa and Neves (2020)	Sport Character Scale (Brazilian version)	N/A	N/A
Rathwell and Young (2016)	University Sport Experience Survey	Internal structure: 605 competitive athletes (Mage = 20.00, SD = 1.74, 237 men)	Internal structure: CFA–9-factor solution–(CFI = 0.911, SRMR = 0.056, RMSEA = 0.040 (90% CI = 0.037–0.043) ESEM: 9-factor solution (CFI = 0.956, SRMR = 0.023, RMSEA = 0.034 (90% CI = 0.029–0.037) Reliability: Cronbach’s α–all factors above: 0.70, except the negative interaction with pears factor: 0.67
Rathwell et al. (2021)	University Sport Experience Scale	Internal structure: 961 university athletes (492 men, Mage = 21.00, SD = 2.89)–divide into two samples for exploratory and confirmatory analyses Test–retest: 24 athletes	Internal structure: ESEM (exploratory sample)–8-factor solution (CFI = 0.930, TLI = 0.886, SRMR = 0.027, RMSEA = 0.044 (90% CI = 0.039–0.048), and *χ*^2^/df = 1.915) ESEM (confirmatory sample)–8-factor solution (CFI = 0.915, TLI = 0.862, SRMR = 0.027, RMSEA = 0.049 (90% CI = 0.045–0.054), and *χ*^2^/df = 2.117) CFA–CFI = 0.904, TLI = 0.895, SRMR = 0.048, RMSEA = 0.043 (90% CI = 0.040–0.047), and *χ*^2^/df = 1.896 Invariance: configural, metric, and scalar invariance across the two sub-samples Test–retest: ICC–Initiative = 0.90; Basic skills = 0.84; Teamwork and Social Skills = 0.78; Adult Networks and Social Capital = 0.71; Stress = 0.90; Social Exclusion = 0.81; Inappropriate Adult Behavior = 0.90, Interpersonal relationships = 0.64 Concurrent validity: ESEM–Coach-athlete relationship had significant positive relationship with initiative (*β* = 0.32), basic skills (*β* = 0.23), interpersonal relationship (*β* = 0.20), teamwork (*β* = 0.29), and adult networks and social capital (*β* = 0.13); and significant negative relationships with stress (*β* = − 0.17), social exclusion (*β* = − 0.24), and inappropriate adult behavior (*β* = − 0.23). All *p*’s < 0.01 Reliability: Construct reliability–Each factor had a CR score above 0.7, except for Basic Skills (CR = 0.66)
Sabourin et al. (2020)	Short Form Youth Experiences Survey (YES-S) and Sport and Life Skills Scale for Sport (LSSS)	Internal structure: 296 students-athletes (Mage = 13.98, SD = 1.31, 215 boys, 6 did not disclose)	Internal structure: CFA–YES-S–5-factor solution–(SB*χ*^2^ = 238.86, df = 199, SB*χ*^2^/df = 1.20, CFI = 0.99, NNFI = 0.99 and RMSEA = 0.026 [0.009–0.037]). LSSS–8-factor solution (SB*χ*^2^ = 1195.43, df = 832, SB-*χ*^2^/df = 1.44, CFI = 0.99, NNFI = 0.99 e RMSEA = 0.039 [0.034–0.043]) Reliability: Cronbach’s α–YES-S–ranged from 0.70 to.80 for all factors, except for the personal and social skills factor: 0.68. LSSS–ranged from 0.77 to 0.92 Test–retest: ICC–YES-S–ranged from 0.43 to 0.56 for all factors, except for the negative experiences factor (0.12). LSSS–ranged from 0.30 to 0.77
Saunders et al. (1997)	The Social Influences scale, The Self-Efficacy scale, The Beliefs scale	422 fifth-grade students (211 boys)	Internal structure: EFA–Social influences scale–1-factor solution. Self-efficacy–3-factor solution. Beliefs–2-factor solution Reliability: Cronbach’s α–Social influences: 0.72, Self-efficacy scale: (support seeking: 0.52; barriers: 0.55; positive alternatives: 0.62), Beliefs scale: (physical outcomes: 0.46; social outcomes: 0.51) Test–retest: ICC–Social influences scale 0.78, Self-efficacy scale (support seeking 0.76, barriers 0.82, positive alternatives 0.61), Beliefs scale (physical outcomes 0.51, social outcomes 0.69)
Sttuntz et al. (2010)	Cross-domain relationship measure—The concept of CDRs was measured using a scale developed by the authors	Internal structure and invariance: 222 athletes (Mage = 14.69, SD = 1.26, 121 boys), 209 collegiate athletes (Mage = 19.99, SD = 1.35, 97 men)	Internal structure: CFA–youth sample (CFI 0.947; TLI = 0.934; RMSEA = 0.073), university students (CFI = 0.947; TLI = 0.934; RMSEA = 0.078) Invariance: MGCFA–configural, metric, and scalar invariance across the two sub-groups (young people and university students) was supported. Residual invariance was not supported Reliability: Cronbach’s α–coach cross-domain relationships (youth sample 0.88, university students 0.85), team mates’ cross-domain relationships (youth sample 0.85, university students 0.90)
Sturm et al. (2020)	Needs Satisfaction in Physical Education Scale (German version)	Internal structure: 507 girls (Mage = 11.61, SD = 0.55)	Internal structure: CFA–3 factors on both levels, not nested (CFI = 0.948; TLI = 0.931; RMSEA = 0.077; SRMR = 0.058) Reliability: Cronbach’s α–for subscales at level 1 ranged from 0.78 to 0.85. Level 2 (class level: 0.79; autonomy: 0.95; competence: 0.18) Composite reliability: for the total scale 0.85 at the individual level and 0.84 for the class level
Vlachopoulos and Michailidou (2006)	The Basic Psychological Needs in Exercise Scale	Internal structure: sample 1–508 exercise participants (Mage = 30.06, SD = 8.13, 254 men; sample 2–504 exercise participants (Mage = 28.92, SD = 8.45, 246 men)	CFA–3-factor solution (CFI = 0.979; NNFI = 0.973; RMSEA = 0.053; SRMR = 0.036) Reliability: Cronbach’s α–autonomy: 0.81; competence: 0.82; relatedness: 0.92 Test–retest: ICC–autonomy: 0.97; competence: 0.97; relatedness: 0.97
Vlachopoulos (2007)	Basic Psychological Needs in Exercise Scale	Internal structure: 851 exercise participants (Mage = 34.16, SD = 10.85, 635 women). Invariance: Sample 1–508 exercise participants (Mage = 30.06, SD = 8.13, 254 women); Sample 2–504 exercise participants (Mage = 28.92, SD = 8.45, 258 women)	Internal structure: CFA–3-factor solution (NNFI = 0.971; CFI = 0.977; RMSEA = 0.061) Invariance: MGCFA–configural, metric, and scalar invariance across participants attending private and community-based fitness centers was supported Reliability: Cronbach’s α–autonomy: 0.84; competence: 0.86; relatedness: 0.92
Vlachopoulos (2008)	Basic Psychological Needs in Exercise Scale in Community Exercise Programs	Invariance: Sample 1–508 exercise participants (Mage = 30.06, SD = 8.13, 254 women). Sample 2–504 exercise participants (Mage = 28.92, SD = 8.45, 258 women) Sample 3–851 exercise participants (Mage = 34.16, SD = 10.85, 635 women)	Internal structure: CFA–men (NNFI = 0.978, CFI = 0.983, RMSEA = 0.051, 90% CI = 0.041–0.061), women (NNFI = 0.984, CFI = 0.987, RMSEA = 0.044, 90% CI = 0.036–0.051) Invariance: MGCFA–configural and scalar invariance and partial metric invariance for sex were supported Reliability: Cronbach’s α–men and women–*α* > 0.70 Composite reliability: men and women– > 0.60
Vlachopoulos et al. (2013)	Basic Psychological Needs in Exercise Scale	Greak: 504 exercise participants (Mage = 28.92, SD = 8.45, 246 men). Spanish: 518 exercise participants (Mage = 29.96, SD = 8.83, 268 men). Portuguese: 989 exercise participants (Mage = 32.67, SD = 10.76, 451 men). Turkish: 686 exercise participants (Mage = 30.78, SD = 8.64, 384 men)	Internal structure: CFA–Greece: CFI = 0.980; RMSEA = 0.042 [90%IC 0.029–0.055], Spain: CFI = 0.906; RMSEA = 0.080 [90% CI 0.069–0.091, Portugal: CFI = 0.954; RMSEA = 0.054 [90% CI 0.046–0.062], Turkey: CFI = 0.912; RMSEA = 0.074 [90% CI 0.064–0.084] Invariance: MGCFA–Greek vs Spanish, Greek vs Portuguese, and Greek vs Turkish (configural and scalar invariance was supported) Reliability: Cronbach’s α–Greece: autonomy: 0.85; competence: 0.80; and relatedness: 0.91. Spain: autonomy: 0.69; competence: 0.77, and relatedness: 0.85. Portugal: autonomy: 0.73, competence: 0.72, and relatedness: 0.83. Turkey: autonomy: 0.78; competence: 0.7; and relatedness: 0.80
Wang et al. (2017)	Self-efficacy for Physical Activity (PASE)	Internal structure: 798 children (between 8 and 13 YO, 445 boys)	Internal structure: EFA–1-factor solution Reliability: Cronbach’s α–0.91 Test–retest: 0.79 IRT (Rating Scale Model): item difficulty, fit indexes (infit/outfit < 1.30), and differential item functioning depending on sex (male and female), age range (8–10 and 11–13 YO), and weight status (underweight, healthy weight, overweight, and obesity) were estimated. Nine items showed dif for one or more variables assessed (varying between 6 items for age range and 2 items for weight status)
Watanabe et al. (2017)	Physical Activity Self-Regulation scale (PASR-12) (Japanese version)	Internal structure: 485 workers (Mage = 42.8, SD = 11.6, 243 men)	Internal structure: CFA–6-factor solution (*χ*^2^ (39) = 100.74, CFI = 0.973, RMSEA = 0.057) Reliability: Cronbach’s α–total score: 0.95; self-monitoring: 0.89; goal setting: 0.89; eliciting social support: 0.90; reinforcement: 0.84; time management: 0.82; relapse prevention: 0.79)
Weiss et al. (1999)	Sport Friendship Quality Scale	Internal structure: 196 sports practitioners (Mage = 11.3, SD = 2.5, 114 boys). Internal structure of revised scale: 194 sports practitioners (Mage = 10.3, SD = 1.2, 168 boys). Internal structure and psychometric properties: 161 sports practitioners (Mage = 10.4, SD = 1.7, 95 boys)	Internal structure: EFA–9-factor solution CFA–6-factor solution (NNFI = 0.93; CFI = 0.94; RMSEA = 0.056) Reliability: Cronbach’s α–self-esteem enhancement and supportiveness: 0.77; loyalty and intimacy: 0.79; things in common: 0.84; companionship and pleasant play: 0.76; conflict resolution: 0.73; conflict 0.91 Test–retest: ICC–self-esteem enhancement and supportiveness: 0.90; loyalty and intimacy: 0.80; things in common: 0.92; companionship and pleasant play: 0.86; conflict resolution: 0.88; conflict 0.87
Weiss et al. (2014)	Life Skill Transfer Survey (LSTS)	Internal structure: 533 youth (Mage = 12.78, range = 10 to 18 YO, 394 boys). Internal structure and reliability: 303 youths from the previous sample (Mage = 13.97, range = 10 to 19 YO, 218 boys)	Internal structure: CFA–second-order model, 8 factors (NNFI = 0.99, CFI = 0.99, RMSEA = 0,059 (90% CI [0.057, 0.062]) Invariance: longitudinal CFA–configural, metric, and scalar invariance across time was supported Reliability: Cronbach’s α–ranged from 0.82 to 0.93 for the eight factors Convergent validity: LSTS vs Youth Experience Survey, convergent validity was supported
Wilson et al. (2006)	The Psychological Need Satisfaction in Exercise Scale	Internal structure: 426 university students (122 men–Mage = 21.38, SD = 2.89; 170 women = Mage = 20.59, SD = 3.02). Sample 2: 581 university students (223 men, Mage = 22.03, SD = 4.16; 358 women, Mage = 21.55, SD = 3.87)	Internal structure: EFA–3-factor solution CFA–total sample (CFI = 0.94; IFI = 0.94; SRMSR = 0.07; RMSEA 0.09), men’s sample (CFI = 0.92; IFI = 0.92, SRMSR = 0.08; RMSEA = 0.09), women1s sample (CFI = 0.93; IFI = 0.93; SRMSR = 0.08; RSMEA = 0.08) Invariance: MGCFA–configural, metric, and scalar invariance across genders Reliability: Cronbach’s α– > 0.90 for all factors

*YO* years old, *mage* mean age, *SD* standard deviation, *PCA* principal components analysis, *CFA* confirmatory factor analysis, *CFI* comparative fit index, *TLI* Tucker–Lewis Index, *RMSEA* root mean square error of approximation, *MGCFA* multigroup confirmatory factor analysis, *SRMR* standardized root mean square residual, *EFA* exploratory factor analysis, *ICC* intraclass correlation coefficient, *ESEM* exploratory structural equation modeling, *CI* confidence interval, *β* beta, *χ*^2^ chi-square, *MGESEM* multigroup ESEM, *α* alpha, *AVE* average variance extracted, *PA* parallel analysis, *WRMR* weighted root mean square residual, *NNFI* non-normed fit index, *GFI* goodness of fit index, *IPLOC* autonomy-internal perceived locus of causality, *TSCI* Trait Sport Confidence Inventory, *CR* construct reliability

The majority of studies originated from the Americas (*n* = 21; 16 from North America and 5 from Brazil), followed by Europe (*n* = 15), Asia (*n* = 5), Africa (*n* = 1), and Oceania (*n* = 2). The total exceeds 41 because some studies were conducted across multiple continents and counted in each corresponding category. The earliest study was published in 1989, followed by two in 1997 and 1999, eight in the 2000 s, and 18 during the 2010 s, indicating a growing trend. Eight studies were published in the current decade (2020s). Regarding the theoretical foundations of the measurement instruments, basic psychological needs (BPN) and Positive Youth Development were the most frequently cited, followed by Structure of Human Values. Other frameworks cited were Emotional Intelligence, Self-efficacy, and Coaching Competence.

Table 2 provides details on the sample characteristics of the studies that conducted content validity procedures. Five studies did not require content validity (Bean et al., 2020; Gunnell et al., 2012; Vlachopoulos, 2007, 2008; Wang et al., 2017) due to one or more of the following reasons: (a) the instrument allowed for contextual wording adaptations without requiring structural modifications or (b) the study focused exclusively on psychometric properties and/or measurement invariance. Among the remaining studies, 15 demonstrated good-quality content validity by involving both experts and the target population. Conversely, 16 studies showed poor-quality content validity: 15 involved only one of the two groups (experts or target population), and one failed to report content validity procedures altogether, despite their necessity. One study, although classified as having poor-quality content validity, consulted experts and used the Flesch-Kincaid readability test in an attempt to ensure age-appropriate item wording, aligning with the aims of readability assessment.

Among the studies included in the review, 13 involved instruments specifically developed within the PYD in sports framework; 11 originated from broader positive development theories and were adapted to PYD in sports; 12 originated from broader positive development theories and were adapted to PDS; and five involved instruments specifically developed for adults/older adults. The most frequently measured constructs were basic psychological needs (15 instruments, 36.58%); life skills (7 instruments, 17.03%); values in sports (3 instruments, 7.31%); and self-efficacy (2 instruments, 4.87%). The remaining constructs listed in Table 2 each represented 2.43% of the instruments. Altogether, the studies included a total of 27,882 participants (≈ 49%, 13,774 boys and men, and ≈ 51%, 14,108 girls and women). For studies focused on physical exercise, the total sample was 9995 (≈ 43%, 4.312 men, and ≈ 57%, 5683 women). For studies focused on sports, the total sample was 17.887 (≈ 53%, 9462 boys and men, and ≈ 47%, 8425 girls and women).

Regarding the type of practice, 43.90% of the studies included individuals from both individual and team sports. A further 21.95% focused on individuals engaged in physical exercise routines, and 12.19% examined school sports settings. Studies exclusively targeting team sports accounted for 9.76%, while 2.43% focused solely on individual sports practitioners, and another 2.43% examined both sports and physical exercise. Two studies (9.76%) did not specify the type of sport or physical exercise, although one instrument was designed for exercise participants. Additionally, 2.43% of the studies focused on content validity procedures, recruiting a small sample of athletes without reporting the specific sport involved.

Most studies employed confirmatory factor analysis (CFA; *n* = 31), exploratory factor analysis (EFA; *n* = 12), or principal component analysis (PCA; *n* = 1) to examine the internal structure of the target instruments. A smaller number used exploratory structural equation modeling (ESEM; n = 4) and Item response theory (IRT; *n* = 1). Reliability was most frequently assessed using Cronbach’s alpha (*n* = 36), followed by composite reliability (*n* = 3). For invariance testing, 11 studies employed multi-group confirmatory factor analysis (MGCFA) while other methods included multi-group exploratory structural equation modeling (MGESEM) (*n* = 1), longitudinal CFA (*n* = 1), structural equation modeling (SEM, *n* = 1), and simultaneous multigroup covariance analyses (*n* = 1).

All 31 studies employing CFA and/or ESEM to test the internal structure reported CFI and root mean square error of approximation (RMSEA) as fit indices, other less reported fit indices were Tucker–Lewis Index (TLI, *n* = 12), standardized root mean square residual (SRMR, *n* = 13), Nonnormed fit index (NNFI, *n* = 10), Goodness of fit index (GFI, *n* = 2), and Incremental fit index (IFI) (*n* = 2). All studies showed acceptable to excellent CFI indices (≥ 0.90) and, with one exception (Myers et al., 2006), all studies reported acceptable to excellent indices for RMSEA (≤ 0.08) (Bentler, 1990). Overall, these findings provide strong evidence supporting the validity of the reviewed instruments for their intended contexts, particularly in terms of their ability to reproduce the original factor structures in adaptation studies.

Twenty-nine studies did not disclose the race/ethnicity breakdown of the samples recruited. Among the 12 studies that disclosed this information, eight included a majority of white people in their sample (range = 54.5% to 91.9%). One study described the sample as predominantly white, while two studies involved samples composed of Chinese and Mexican participants, respectively (Laffrey & Asawachaisuwikrom, 2001; Wang et al., 2017).

## Discussion

The objective of this systematic review was to identify measurement instruments for positive development in sports, the theoretical foundations underpinning these instruments, and the validity evidence supporting their use. The findings provide both the academic and professional communities with an overview of available PDS instruments and their key characteristics. The most frequently examined types of validity evidence were internal structure and internal consistency, both of which strongly supported the quality of these measures in assessing the intended constructs (American Educational Research Association [AERA] et al., 2014). Findings indicate that the instrument items and components were generally aligned with their theoretical foundations, effectively capturing the proposed constructs.

For example, Albouza et al. (2021) adapted the Youth Sport Values Questionnaire (YSVQ-2), to assess values in sport, focusing on three subscales: morality, competence, and status. Reported fit indices (CFI/TLI ≥ 0.90; RMSEA ≤ 0.08) suggest that the model fits the sample data well, confirming that each subscale reliably measures values in sport. Additional analyses, such as Cronbach’s alpha coefficients, indicated high internal consistency in estimating respondent scores on the subscales, further supporting the validity of the instrument (Albouza et al., 2021; Cunha et al., 2016). Similar findings by Lee et al. (2008) and Gonçalves et al. (2017) reinforce the robustness of the YSVQ-2. This consistency across studies, time periods, and diverse samples supports both the reliability and applicability of these instruments (AERA, 2014). A similar pattern was observed in studies on the Basic Psychological Needs in Exercise Scale, further affirming the stability of validity evidence in this field.

The use of indices such as CFI, TLI, and RMSEA is both appropriate and expected when assessing internal structure. CFI and TLI compare the proposed model to a null model while penalizing for model complexity (with widely accepted cutoffs of ≥ 0.90), and RMSEA estimates the approximation error between the model and the observed data (with a recommended cutoff of ≤ 0.08) (Brown, 2015; Tabachnick & Fidell, 2019). However, merely reporting these fit indices does not ensure construct validity. Complementary statistical evidence that support the model along with a solid theoretical foundation are also essential criteria.

Internal consistency, typically assessed using Cronbach’s alpha, also warrants closer scrutiny. Although widely used, this index is highly sensitive to the number of items in a factor, which can artificially inflate the results without truly reflecting the construct homogeneity (Doval et al., 2023; Pasquali, 2017). Overall, the instruments included in this review demonstrated good fit indices and high reliability, contributing with valuable tools for assessing PDS within sports contexts. However, the lack of alternative reliability estimates, such as McDonald’s omega or composite reliability, which are more appropriate for hierarchical models, reveals a significant methodological gap (Doval et al., 2023).

Sport has long been regarded as a fertile environment for fostering positive development, given its potential to support psychological and social growth (Aoyagi et al., 2012; Carvalho, 2020). The findings of this review provide researchers and practitioners with resources to evaluate interventions and better understand how sports can promote PDS. However, no studies addressing the development of interpretative norms were identified. This limits the practical application of these instruments, as they lack reference parameters for interpreting raw scores.

Another critical issue is the scarcity of measurement invariance testing, which is essential to ensure that instruments function equivalently across different groups. Of the 41 studies analyzed, only nine conducted such analyses, and none addressed dimensions such as race or ethnicity. This omission undermines the validity of subgroup comparisons and reflects a lack of attention to issues of social justice in measurement practices (AERA et al., 2014; Diemer et al., 2019). Ignoring these dimensions can contribute to the perpetuation of inequalities, as psychological instruments are embedded with cultural assumptions that must be critically examined. Future studies are encouraged to adopt more inclusive recruitment strategies, use detailed sociodemographic questionnaires that incorporate race and ethnicity, and apply measurement invariance analyses, such as multigroup confirmatory factor analysis or multiple indicators and multiple causes (MIMIC) models, to ensure that instruments are equivalent across groups and produce valid inferences.

Almost half of the studies included in this review focused on developing new instruments, while the other half focused on adapting existing instruments for different cultures or contexts. Most instruments were developed in high-income countries, and only five adaptation studies were conducted in middle-income countries, highlighting a significant geographic imbalance. Positive Youth Development originated in North America (Larson, 2000), where it has been extensively studied in high-income countries such as the USA and Canada. However, Patton et al. (2009) reported that 89% of the world’s youth (aged 10–24 years) live in low- and middle-income countries, where youth experiences often differ from that of their counterparts living in wealthier nations due to more profound challenges such as poverty, limited access to healthcare, and educational inequality. This disparity underscores the need for future research to develop instruments tailored to these populations, while also revisiting and revising the theoretical foundations of existing tools to ensure contextual relevance.

Regarding age groups, most instruments targeted children, adolescents, and young adults in the sports context. Fewer instruments focused on adults and older adults, and more instruments were designed for use in physical exercise settings rather than for younger athletes. Some studies included participants across different life stages, such as Aguilar and Petrakis (1989), who examined individuals aged 18–74 years. The concentration of instruments on younger people reflects the origins of PDS in youth sports. Nonetheless, the literature highlights that the positive development is equally relevant for older cohorts, based on the premise that human potential can be cultivated at any age. Indeed, PDS has been explored in various age groups, including young adults (Rathwell & Young, 2019) and older adults (Baker et al., 2010). Furthermore, sport is widely regarded as an activity conducive to personal development (Korsakas et al., 2021; Larson, 2000). Yet, because human development has traditionally been associated with childhood and adolescence, the developmental potential of sports for adults and older adults remains underappreciated (Baker et al., 2010). Alternative perspectives on human development suggest that individuals continue to learn and grow throughout later life stages (Dionigi et al., 2017; Gergen & Gergen, 2001). Therefore, the limited availability of instruments assessing PDS for adults, and even fewer for older adults, represents a gap that future research should address.

Kim et al. (2019), in a systematic review, highlighted the lack of instruments addressing older cohorts. They concluded that psychological and social outcomes of sports participation among older adults remain largely unexplored and recommending the development of new instruments for this demographic. Although some tools exist for adults, greater investment is needed, particularly in constructing instruments for adults in low- and middle-income countries. Notably, only two studies included participants aged 60 and older, further underscoring the underrepresentation of this group in sports research.

It is well documented that girls and women are a disadvantaged group in sports, facing fewer opportunities to participate, exposure to prejudice and violence once involved, and access to lower-quality training and competition environments (Korsakas et al., 2024; Maranhão et al., 2023). Therefore, it is imperative to ensure that professionals and researchers have access to high-quality strategies for measuring the effects of interventions in sports contexts, enabling data collection that can inform practice and guide planning. Current best practices for developing and adapting measurement instruments recommend adopting practices to prevent bias, including accounting for the specific experiences of different groups, particularly those who are minoritized (Diemer et al., 2019). In recognition of this issue, we conducted an analysis of the gender breakdown in the samples of the studies included in this review.

Gender analysis revealed relatively balanced overall participation, with slightly more girls and women (*n* = 14,108) than boys and men (*n* = 13,774). Regarding the type of activity, there were more women (*n* = 5683) than men (*n* = 4312) in physical exercise settings, while sports-related studies included more men (*n* = 9462) than women (*n* = 8425). These results are consistent with cultural patterns, as physical exercise is more widely accepted for women, whereas sport is still often perceived as a masculine domain (Korsakas et al., 2024). Despite this, most studies included a relatively equal gender distribution, with 9 out of 15 conducting invariance tests that incorporated gender as a variable. Additionally, three studies reported individuals identifying as gender fluid, suggesting a growing acknowledgment of gender diversity in the development and adaptation of measurement instruments, an area warranting further research investment.

When analyzing race/ethnicity data, most studies did not report these characteristics. Only 12 of the 41 studies reviewed reported race/ethnicity information and eight predominantly included white participants. These findings highlight the need for studies on instrument development and adaptation to explicitly account for race/ethnicity when recruiting participants, particularly in sport contexts. Reporting this information is essential for professionals and researchers to determine whether an instrument is suitable for their specific population. Such practices can support the development of more equitable sports initiatives that address the needs of racial and ethnic minoritized groups.

Most instruments reviewed were based on theoretical models linked to the development of positive characteristics but were not explicitly constructed within the PDS framework or designed primarily for evaluating athletes. This is evident in adaptations such as the Youth Experiences Survey-YES (MacDonald et al., 2012) and the Psychological Need States in Sport Scale (Bhavsar et al., 2020), which required revisions to their internal structures when applied in the sports context. To address this limitation, it is recommended that new instruments be developed based on robust theoretical models of PDS. This approach would ensure that the constructs are properly operationalized and that the instruments’ factorial structures are conceptually aligned with the intended framework.

This systematic review contributes by presenting available tools for assessing positive development in sports and identifying key areas that would benefit from further research. Limitations include potential omissions due to the combinations of terms used in the search strategy and databases used, even knowing we carefully developed and revised the search strategy. While the review offers a broad overview of instruments, their main characteristics and psychometric properties, it does not deeply evaluate the quality of psychometric analyses, which would aid professionals in selecting appropriate tools. For example, current academic discussions emphasize the importance of using robust estimators for assessing internal structure of instruments that adapt to the characteristics of the items (Li, 2016) and for assessing reliability (Doval et al., 2023). Therefore, we encourage future studies to address this topic and recommend readers to analyze it thoroughly. Another limitation is the absence of a formal risk of bias assessment for included studies according to COSMIN guidelines. It was not our objective to identify the most appropriate instrument, since this depends on the context, therefore, we decided to assess studies quality based on international standards (American Educational Research Association et al., 2014) and include them even if the quality of validity evidence reported was poor, which helped us to indicate future directions for the field.

## Conclusion

This systematic review identified key opportunities to advance the field and provides directions for future research. Many existing instruments are based on broad frameworks, such as basic psychological needs, which are not explicitly rooted in the positive development in sport (PDS) framework. Developing instruments grounded in specific theoretical models is essential to accurately assessing distinct dimensions of PDS, thereby enhancing their precision and applicability.

The predominance of instruments focused on youth highlights a significant gap in the development of tools for older groups. Considering that human potential can be cultivated throughout life, it is essential to develop instruments that assess the psychological and social benefits of sports across different life stages. Additionally, most instruments originated in high-income countries, mainly in North America and Europe, underscoring the need to develop tools suited to low- and middle-income contexts. This is essential to promote broader applicability and ensure equity in sports practices.

Furthermore, future studies should consider constructing instruments that are theoretically grounded in positive development in sports, which will bring theoretical robustness to the instrument and contribute to additional development in theory. Researchers should also avoid the uncritical importation of theories without conducting necessary cross-cultural adaptations. Such adaptations require meaningful engagement with the target population within its specific cultural context. Theories must be examined within local cultures before measurement instruments are developed, particularly given the substantial cultural differences between developed and developing countries. It is equally important to prioritize diverse populations in terms of age group, gender, race/ethnicity, and gender identity when constructing new instruments or adapting them to different contexts. This includes not only adopting recruitment strategies that intentionally include underrepresented populations, but also ensuring that underlying theories adequately reflect their experiences. Instruments that consider these differences are essential for designing sports interventions that are both effective and socially representative. Moreover, the absence of interpretative norms limits the practical utility of existing instruments. Investing in the development of clear and culturally sensitive norms will enable professionals and researchers to plan and evaluate interventions with greater confidence and contextual relevance.

## Data Availability

All data generated or analyzed during this study are included in this published article.

## Abbreviations

PDS: Positive development in sports
PYD: Positive Youth Development
5Cs: Five Cs of positive development
4Cs: Four Cs of positive development
BPN: Basic psychological needs
CFA: Confirmatory factor analysis
EFA: Exploratory factor analysis
ESEM: Exploratory structural equation modeling
IRT: Item response theory
MGCFA: Multi-group confirmatory factor analysis
MGESEM: Multi-group exploratory structural equation modeling
SEM: Structural equation modeling
CFI: Comparative fit index
RMSEA: Root mean square error of approximation
TLI: Tucker–Lewis index
NNFI: Normed fit index
GFI: Goodness-of-fit index
IFI: Incremental fit index
YSVQ-2: Youth Sport Values Questionnaire-2
YES: The Youth Experiences Survey
YO: Years old
W: Women
M: Men
N/A: Not applicable
CO: Construction
TA: Transcultural adaptation
AD: Adaptation
CI: Cross-cultural invariance
VA: Validity evidence accumulation
SD: Standard deviation
PCA: Principal components analysis
SRMR: Standardized root mean square residual
ICC: Intraclass correlation coefficient
CI: Confidence interval
β: Beta
χ2: Chi-square
α: Alpha
AVE: Average variance extracted
PA: Parallel analysis
WRMR: Weighted root mean square residual
NNFI: Non-normed fit index
GFI: Goodness of fit index
IPLOC: Autonomy-internal perceived locus of causality
TSCI: Trait sport confidence inventory
CR: Construct reliability
